# Baseline Choroidal Blood Flow Imbalance as a Predictive Factor for Macular Edema Recurrence Secondary to Branch Retinal Vein Occlusion

**DOI:** 10.3390/diagnostics14202328

**Published:** 2024-10-18

**Authors:** Ryuya Hashimoto, Kenichiro Aso, Keisuke Yata, Naoki Fujioka, Kazufumi Tanaka, Serika Moriyama, Asato Hirota, Juri Kawamura, Takatoshi Maeno

**Affiliations:** Department of Ophthalmology, Toho University Sakura Medical Center, 564-1, Shimoshizu, Sakura 285-8741, Japantakatoshi.maeno@ophthal.med.osaka-u.ac.jp (T.M.)

**Keywords:** laser speckle flowgraphy, branch retinal vein occlusion, macular edema, anti-VEGF drugs, choroidal blood flow, choroidal thickness

## Abstract

Background/Objectives: To evaluate the roles of choroidal blood flow (CBF) and choroidal thickness (CT) as predictors of macular edema recurrence in patients with treatment-naive non-ischemic branch retinal vein occlusion (BRVO) after intravitreal ranibizumab (IVR) injection. Methods: Sixteen eyes from sixteen patients with treatment-naive non-ischemic BRVO treated with IVR, once initially and then as needed, were included in the study. CBF and CT in the subfovea, occlusive, and non-occlusive regions were measured via laser speckle flowgraphy and enhanced depth imaging optical coherence tomography over 12 months. Results: Baseline CT was significantly greater in the occlusive region (335 ± 72.1 µm) than in the non-occlusive region (274 ± 36.7 µm, *p* = 0.028). CT in the occlusive region was reduced significantly after 1 week (*p* = 0.008), but CBF did not change significantly after IVR throughout the follow-up period (*p* > 0.05). The occlusive/non-occlusive region CBF ratio at baseline was significantly associated with the number of IVR injections over 12 months (mean 2.63) in patients with BRVO (*p* = 0.048). Conclusions: Baseline CBF imbalance in eyes with treatment-naive BRVO may indicate the recurrence of macular edema after ranibizumab therapy.

## 1. Introduction

Branch retinal vein occlusion (BRVO) is the most common cause of retinal vascular disease. Macular edema (ME) is a major complication and is frequently associated with reduced visual acuity (VA) in patients with BRVO [1]. Previous studies have demonstrated that ME associated with BRVO was significantly reduced by anti-vascular endothelial growth factor (VEGF) treatment [2,3]. However, a major problem with anti-VEGF therapy is the frequent recurrence of ME, which necessitates repeated injections. Thus, it is important to identify factors that predict the recurrence of ME early in the course of BRVO.

There are several previous reports of changes in choroidal thickness (CT) [4,5,6] and ocular circulation [7,8,9] in eyes with BRVO. Kim et al. [10] demonstrated that CT in the affected eye was greater in the occlusive region than in the non-occlusive region using enhanced depth imaging optical coherence tomography (EDI-OCT) [11]. Subfoveal CT was also reported to be associated with response to intravitreal ranibizumab (IVR; Lucentis, Genentech, South San Francisco, CA, USA) injection throughout the 6-month study period [6]. Regarding ocular blood flow, Nagaoka et al. [7] reported that retinal blood flow before anti-VEGF therapy may be a significant predictor of response (over 6 months) in patients with BRVO secondary to ME.

However, there have been no reports of time-course changes in choroidal blood flow (CBF) and CT in patients with treatment-naive BRVO after IVR treatment throughout long-term follow-up.

The primary aims of the current study were to investigate changes in CBF and CT in the occlusive and non-occlusive regions before the administration of IVR therapy and over 12 months after the initiation of IVR therapy. CBF and CT were assessed via laser speckle flowgraphy (LSFG-NAVITM, Softcare Co., Ltd., Fukuoka, Japan), which can measure choroidal circulation [12,13,14,15,16], and EDI-OCT. We also examined potentially predictive factors regarding the recurrence of ME secondary to treatment-naive non-ischemic BRVO.

## 2. Materials and Methods

### 2.1. Participants

Sixteen eyes from sixteen subjects (ten male and six female, mean age 67.6 ± 13.2 years) with non-ischemic BRVO who had not received any treatment were consecutively enrolled in this retrospective analysis study. The study was conducted at Toho University Sakura Medical Center from September 2015 to July 2016. All procedures were in full compliance with the guidelines of the Declaration of Helsinki and were approved by the Institutional Review Board/Ethics Committee of Toho University Sakura Medical Center (number: 2015-056).

Patients were eligible for inclusion if they had ME associated with treatment-naive non-ischemic BRVO, were evaluated over 12 months, were enrolled within 12 weeks of BRVO onset, if the affected eye had a decimal best-corrected visual acuity (BCVA) ≧ 0.3 and a central foveal thickness (CFT) > 300 µm, central fovea involvement was apparent via optical coherence tomography (OCT; Spectralis OCT^®^, Heidelberg Engineering Inc., Heidelberg, Germany) at the initial study visit, and symptom duration was <6 months prior to examination. BCVA is a critical parameter used to quantify the sharpest level of vision achievable by an individual when wearing appropriate corrective lenses. Decimal BCVA can be calculated based on the following formula: Decimal BCVA = 10^−logMAR^.

The diagnosis of BRVO was based on fundus examination and fluorescein angiography (FA) findings and was determined by retinal specialists (R.H., K.A., K.Y., N.F., K.T., S.M., and T.M.).

Patients were excluded if they had glaucoma, optic neuropathy, another chorioretinal disease (e.g., diabetic retinopathy, uveitis, age-related macular degeneration, epiretinal membrane, or macular hole), a history of previous vitreous surgery or photocoagulation, high myopia (refractive error worse than −6 D), a history of intravitreal injection of anti-VEGF or triamcinolone acetonide, or a history of taking anticoagulants, antiplatelet agents, or corticosteroids. These conditions or treatments were excluded as they could potentially influence ocular blood flow and confound the results of the study, making it difficult to accurately assess the effects of the interventions being evaluated. During the initial visit, we performed FA prior to the IVR injection. Where the non-perfusion area was greater than 6 optic disc areas, we defined the case as ischemic BRVO and such cases were excluded.

### 2.2. Treatment Protocol for Intravitreal Ranibizumab (IVR) Injections and Management of Macular Edema

All 16 eyes received one initial IVR injection, and additional injections were administered pro re nata when the CFT remained > 300 µm and intraretinal fluid was apparent on OCT examination. In cases of ME recurrence, we administered an IVR injection on the day of recurrence.

### 2.3. Examinations

During follow-up, patients received ocular examinations every month after IVR for 12 months; the results at day 1, week 1, and months 1, 2, 3, 6, 9, and 12 were analyzed. The examinations included BCVA measurement, slit-lamp assessment, fundus photography, OCT, EDI-OCT, and LSFG. Six months after the initial visit, we performed an FA examination again to determine whether individual patients in the study had developed ischemic BRVO. BCVA was measured as decimal VA using a Landolt C chart and converted to the logarithm of minimal angle of resolution (logMAR).

### 2.4. Measurement of Choroidal and Retinal Thickness

**Figure 1:**
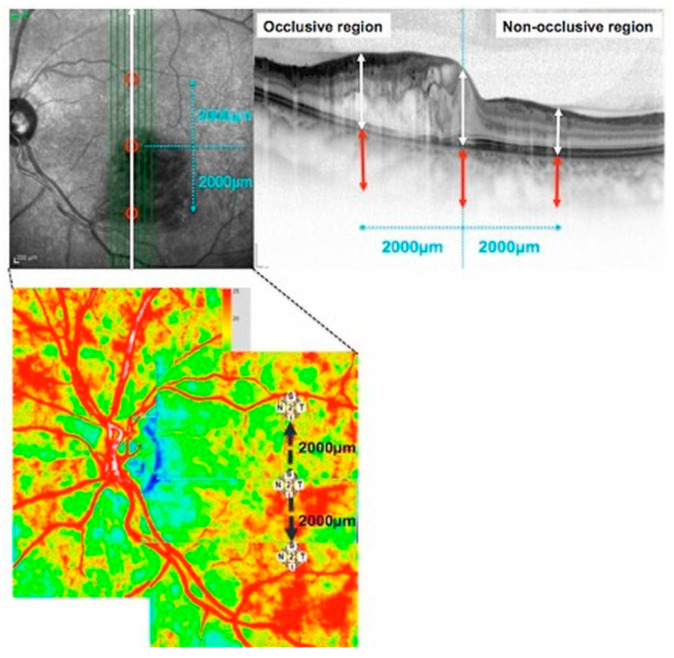
Measurement of retinal thickness, choroidal thickness, and choroidal blood flow using enhanced depth imaging optical coherence tomography and laser speckle flowgraphy. **Top left**: OCT en face image of the fundus with green scan lines indicating B-scan location. **Top right**: OCT B-scan showing occlusive (left) and non-occlusive (right) regions. White arrows indicate retinal thickness measurements; red arrows indicate choroidal thickness measurements. **Bottom**: Laser speckle flowgraphy (LSFG) panorama map; red areas represent higher blood flow, blue areas indicate lower blood flow. Measurements were taken at the central macula and at points 2000 μm superior and inferior to the center, similar to the OCT measurements and corresponding to the scale bar shown.

Three independent examiners (A.H. and J.K.) measured the retinal thickness and choroidal thickness (CT) at the central fovea (subfovea), 2000 µm superior to the fovea, and 2000 µm inferior to the fovea using vertical scan images (Figure 1, top left). Where the artery-vein crossing was located 2000 µm superior or inferior to the fovea, we classified the region as occlusive. We defined the occlusive region as the area at arteriovenous crossings, typically marked by hemorrhage and compromised venous outflow due to BRVO. Conversely, we defined the non-occlusive region as the opposite side, where no arteriovenous crossings or hemorrhage were present, and venous outflow remained intact. These classifications were used for comparative analyses of CBF and retinal thickness in the affected and unaffected regions.

CT was determined via EDI-OCT by manually measuring the distance from the outer border of the hyper-reflective line corresponding to the retinal pigment epithelium (RPE) to the outer border of the choroid beneath the fovea, using a horizontal scan through the fovea (Figure 1, top right). All examinations were performed between 12:00 noon and 3:00 p.m. to avoid circadian variations in CT [17].

### 2.5. Measurement of Choroidal Blood Flow Using Laser Speckle Flowgraphy

In a previously reported case study, the LSFG-NAVI^TM^ system (Softcare Co., Ltd., Fukuoka, Japan) was useful in assessing choroidal [12,13,14,15,16] and optic nerve head circulation.

In this study the same LSFG system was used to examine choroidal circulation in patients with treatment-naive non-ischemic BRVO. The methods used to assess ocular blood flow via LSFG have been described in detail previously [18,19]. In this study, LSFG was used to measure the mean blur rate (MBR) as an indicator of blood flow [20].

The circle size used for measuring CBF was initially defined based on the macular region’s foveal avascular zone (FAZ). To ensure accuracy, LSFG images were overlaid on fluorescein angiography (FA) images using LSFG Analyzer software (Ver. 3.5.0.0, Softcare Co., Ltd., Fukuoka, Japan). The measurement circle was first placed within the FAZ to avoid interference from retinal vasculature, and this same circle size was subsequently applied to both the occlusive and non-occlusive regions for consistency. To evaluate the time-course of CBF in the three regions investigated (subfoveal, occlusive, and non-occlusive), the measurement circle was set at these regions at each time-point (Figure 1, bottom left). The position of each circle was manually determined by comparing fundus photographs with LSFG color map images.

If retinal vessels overlap the LSFG measurement area, this does not significantly affect choroidal blood flow (CBF) measurements. As Isono et al. demonstrated, LSFG primarily visualizes choroidal circulation, with approximately 92% of the measured flow being choroidal, even in areas with both retinal and choroidal vessels [12].

MBRs were measured three times at each point and used to calculate an average MBR value. Thirty minutes before LSFG measurement, mydriasis was induced via the administration of eye drops containing 0.5% tropicamide and 0.5% phenylephrine hydrochloride (Mydrin-P ophthalmic solution, Santen Pharmaceutical Co., Ltd., Osaka, Japan). LSFG measurements were obtained after the subject had rested for 10 min in a quiet room maintained at 24 °C with mydriasis to stabilize ocular hemodynamics; this ensured consistent measurements and reduced potential variability due to room temperature changes. We measured CBF three times and used the average of these three measurements for statistical analysis. To evaluate the time-course of CBF at the three regions investigated, we calculated and analyzed MBR values from the choroid using LSFG Analyzer software (Ver. 7.1.79.0, Softcare Co., Ltd., Fukuoka, Japan). The MBR is a quantitative index that represents relative CBF values, i.e., the percentage compared with the baseline. We also calculated the occlusive/non-occlusive region CBF ratio derived from the relative values before and after IVR. The MBRs were used to calculate the occlusive/non-occlusive region CBF ratio. The MBRs were also calculated as a percentage relative to the baseline to investigate the time-course of CBF.

### 2.6. Evaluation of Ocular Hemodynamics

It was previously reported that there is a linear relationship between CBF and ocular perfusion pressure (OPP) [21]. Intraocular pressure (IOP) and systolic blood pressure (SBP)/diastolic blood pressure (DBP) were measured at the same time as CBF. All these measurements were performed in the seated position. Mean blood pressure (MBP) was calculated from the SBP and DBP values using the following equation:

MBP = DBP + 1/3 (SBP − DBP)

The following equation was used to calculate OPP [22]:

OPP (seated position) = (2/3 × MBP) − IOP

### 2.7. Statistical Analysis

All results are expressed as the mean ± standard deviation (SD), and *p* < 0.05 was deemed to indicate statistical significance. The Shapiro–Wilk test was used to assess the normality of distributions. Changes in VA, retinal thickness, CT, and CBF from baseline were assessed by performing a one-way repeated measures analysis of variance (ANOVA) and post hoc Bonferroni correction. A repeated measures ANOVA was used to examine differences in the clinical course of retinal thickness, CT, and CBF in each region. Pearson’s correlation coefficients were calculated to assess associations between the quantitative variables. All statistical analyses were conducted using SPSS version 23 (IBM Corp., Armonk, NY, USA).

## 3. Results

### 3.1. Clinical and Laboratory Characteristics of Patients with Non-Ischemic Branch Retinal Vein Occlusion

Table 1 summarizes the clinical and laboratory characteristics of the 16 patients in the study (10 male and 6 female, mean age 67.6 ± 13.2 years, 10 major BRVO and 6 macular BRVO). The mean BRVO duration was 7.8 ± 8.0 weeks (Table 1). Systemic diseases were present among the patients, including hypertension (13/16, 81.3%), hyperlipidemia (7/16, 43.8%), and type 2 diabetes mellitus (1/16, 6.3%).

### 3.2. Time-Course of Best-Corrected Visual Acuity

The mean baseline logMAR VA was 0.32 ± 0.23, and after IVR, it had improved to 0.25 ± 0.21, 0.15 ± 0.17, 0.05 ± 0.12, 0.09 ± 0.19, 0.07 ± 0.14, 0.06 ± 0.16, 0.04 ± 0.22, and 0.06 ± 0.17 at day 1, day 7, and months 1, 2, 3, 6, 9, and 12, respectively (all *p* < 0.05 vs. baseline, one-way repeated ANOVA with Bonferroni correction).

### 3.3. Baseline Retinal and Choroidal Thickness in Eyes with Branch Retinal Vein Occlusion

**Figure 2:**
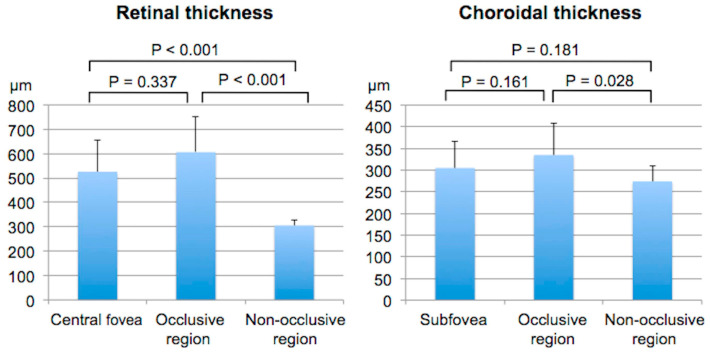
Baseline mean retinal and choroidal thicknesses in eyes with branch retinal vein occlusion.

The mean retinal thicknesses in the central fovea, occlusive region, and non-occlusive region in the affected eyes are shown in Figure 2 (left). The mean retinal thickness in the occlusive region (606 ± 148 µm) was significantly greater than in the non-occlusive region (305 ± 24.7 µm; *p* < 0.001) but not in the central fovea (526 ± 129 µm; *p* = 0.337). Mean CTs in the subfovea, occlusive region, and non-occlusive region at baseline are shown in Figure 2 (right). The mean CT in the occlusive region (335 ± 72.1 µm) was significantly greater than in the non-occlusive region (274 ± 36.7 µm; *p* = 0.028) but not in the subfovea (305 ± 60.7 µm; *p* = 0.161).

**Figure 3:**
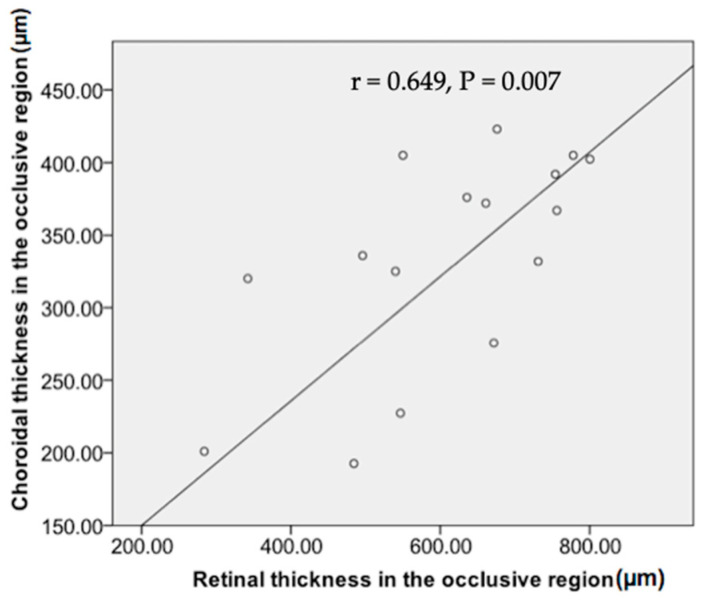
Correlation between choroidal thickness and retinal thickness in the occlusive region in 16 eyes affected with branch retinal vein occlusion.

The mean CT in the occlusive region was significantly positively correlated with mean retinal thickness in the occlusive region at baseline (r = 0.649, *p* = 0.007, Pearson’s correlation analysis; Figure 3)

### 3.4. Time-Course of Retinal and Choroidal Thicknesses After Intravitreal Ranibizumab Injection Therapy in Eyes with Branch Retinal Vein Occlusion

**Figure 4:**
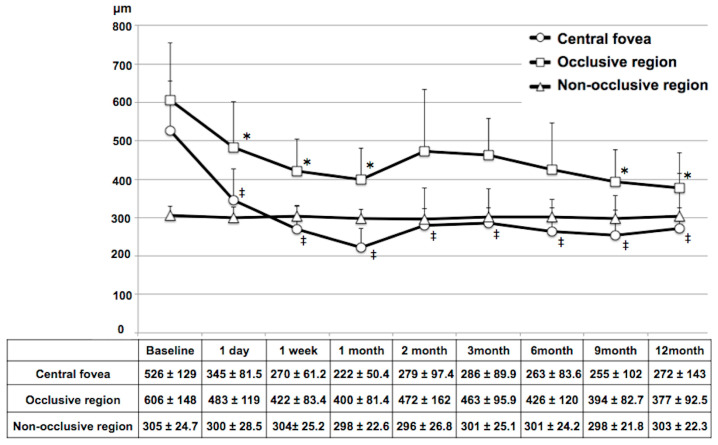
Time-course of retinal thickness after intravitreal ranibizumab injection therapy in eyes with branch retinal vein occlusion. * Statistically significant difference compared to baseline in the occlusive region. ‡ Statistically significant difference compared to baseline in the central fovea. (*p* < 0.05 following Bonferroni correction).

The time-course of mean retinal thickness after IVR over a 12-month period is shown in Figure 4. The mean retinal thickness in the central fovea was 536 ± 129 µm at baseline, and it was significantly lower than this at every time-point assessed during the follow-up period (all *p* < 0.05). The mean retinal thickness in the occlusive region was 606 ± 148 µm at baseline, and it was significantly lower than this at the 1-day, 1-week, and 1-, 9-, and 12-month follow-up time-points (all *p* < 0.05). The mean retinal thickness in the non-occlusive region did not change significantly during the follow-up period.

**Figure 5:**
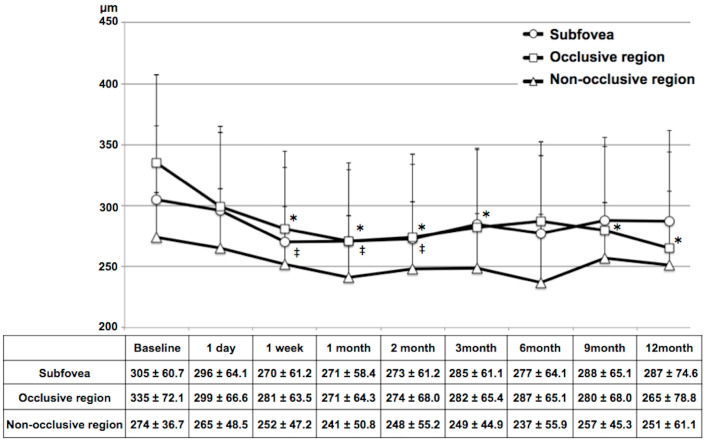
Time-course of choroidal thickness after intravitreal ranibizumab injection therapy in eyes with branch retinal vein occlusion. * Statistically significant difference compared to baseline in the occlusive region. ‡ Statistically significant difference compared to baseline in the subfovea. (*p* < 0.05 following Bonferroni correction).

The time-course of mean CT after IVR over a 12-month period is shown in Figure 5. The mean CT in the subfovea was 305 ± 60.7 µm at baseline, and it was significantly lower than this at the 1-week, 1-month and 2-month follow-up time-points (all *p* < 0.05). The mean CT in the occlusive region was 335 ± 72.1 µm at baseline, and it was significantly lower than this at the 1-week, and 1-, 2-, 3-, 9-, and 12-month follow-up time-points (all *p* < 0.05). The mean CT in the non-occlusive region did not change significantly during the follow-up period.

### 3.5. Time-Course of Choroidal Blood Flow After Intravitreal Ranibizumab Therapy in Eyes with Branch Retinal Vein Occlusion

**Figure 6:**
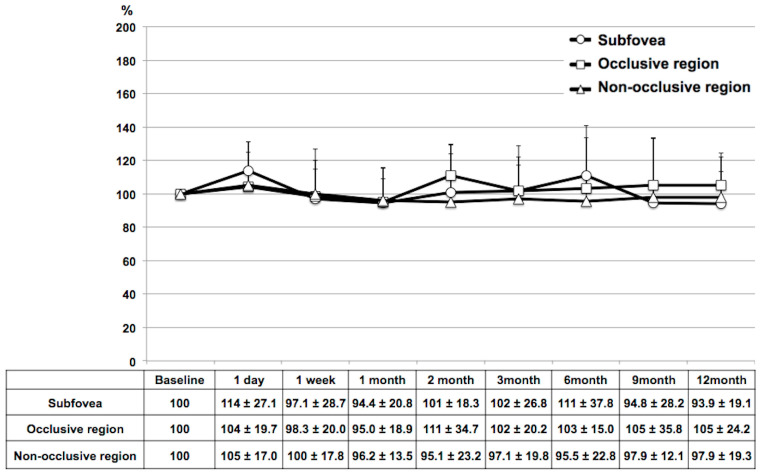
Time-course of choroidal blood flow after intravitreal ranibizumab injection therapy in eyes with branch retinal vein occlusion.

The time-course of mean CBF (%) in each region after IVR over a 12-month period is shown in Figure 6. The mean CBF in the subfoveal, occlusive, and non-occlusive regions was 10.3 ± 6.4/9.7 ± 3.3/10.2 ± 3.8, respectively, at baseline, and it did not differ significantly from baseline at the 1-day (10.9 ± 5.2/9.7 ± 2.8/10.5 ± 3.6), 1-week (9.1 ± 3.7/9.3 ± 3.2/9.9 ± 43.3), 1-month (9.2 ± 4.4/9.0 ± 2.9/9.4 ± 2.7), 2-month (10.1 ± 4.4/10.3 ± 3.3/9.2 ± 2.4), 3-month (9.8 ± 4.7/9.7 ± 3.3/9.5 ± 2.8), 6-month (9.7 ± 2.8/9.8 ± 2.8/9.5 ± 3.6), 9-month (8.8 ± 3.6/10.6 ± 2.7/10.0 ± 3.9), or 12-month (9.0 ± 3.8/10.0 ± 3.7/9.6 ± 2.9) follow-up time-points.

### 3.6. Hemodynamics

The mean OPP was 56.5 ± 9.2 mmHg at baseline, and it did not differ significantly from baseline at the 1-day (57.2 ± 8.0 mmHg), 1-week (57.4 ± 12.1 mmHg), 1-month (56.9 ± 6.9), 2-month (57.7 ± 6.8 mmHg), 3-month (56.5 ± 8.1), 6-month (56.9 ± 5.8), 9-month (55.4 ± 5.8), or 12-month (57.1 ± 7.9 mmHg) follow-up time-points.

Factors related to the mean number of macular edema recurrences in patients with branch retinal vein occlusion.

**Figure 7:**
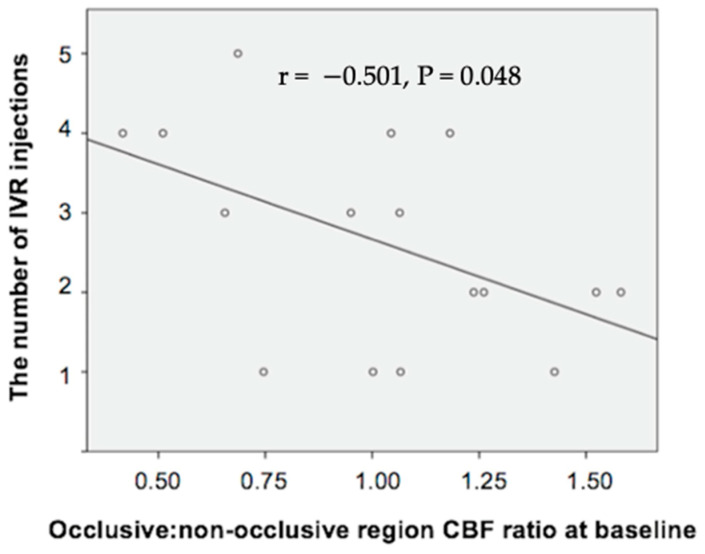
Correlation between the number of IVR injections and the occlusive to non-occlusive region choroidal blood flow ratio at baseline.

We analyzed the relationships between the mean number of IVR injections for ME recurrence over the 12 months (2.63 ± 1.27, range 1–5) and the other variables investigated (Table 2). Pearson’s correlational analysis of the dependent variables showed that the mean number of IVR injections was significantly positively correlated with logMAR VA (r = 0.589, *p* = 0.016) and CFT (r = 0.697, *p* = 0.0013) at baseline. Furthermore, the number of IVR injections was significantly negatively correlated with the occlusive/non-occlusive region CBF ratio at baseline (r = −0.501, *p* = 0.048) among the subjects (Table 2, Figure 7).

**Figure 8:**
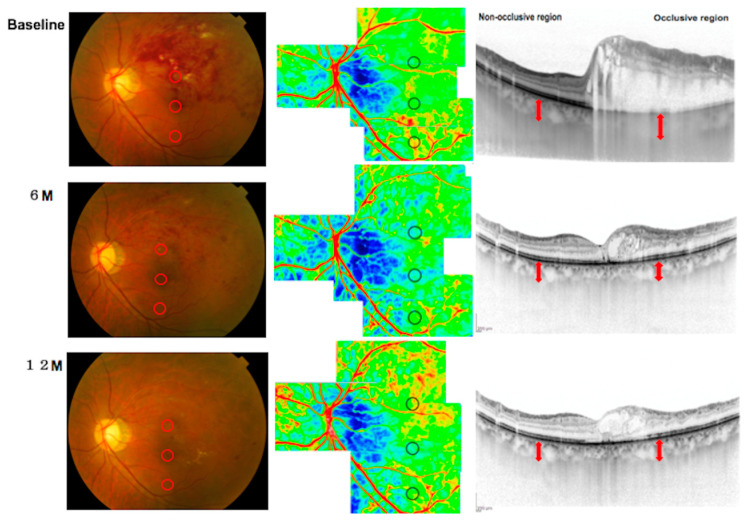
Representative images of fundus photography, laser speckle flowgraphy (LSFG), and enhanced depth imaging optical coherence tomography (EDI-OCT) in a patient with lower occlusive to non-occlusive region choroidal blood flow ratio. The left panel displays a fundus photograph of the affected eye, with the occlusive region located superiorly. Red circles highlight points 2000 µm superior and inferior to the macular center. The middle panel shows the LSFG composite panorama map, with black circles marking the same locations 2000 µm from the macula. The right panel presents OCT B-scans, with the occlusive region appearing on the right side of the scan. Choroidal thickness measurements are indicated by red arrows. All measurements were performed at the macula and at points 2000 µm superior and inferior to the macular center, ensuring consistency across the OCT and LSFG modalities.

Figure 8 shows a representative case of fundus photography, LSFG, and EDI-OCT findings in patients with a major BRVO at the baseline, 6-, and 12-month follow-up periods. The case is a 66-year-old man with a major branch retinal vein occlusion in the left eye. The best-corrected decimal visual acuity is 0.3. He received an intravitreal injection of ranibizumab (IVR) five times during the 12-month follow-up period. Before treatment, the occlusive/non-occlusive region CBF ratio was 0.68 (9.1/13.3), and the CT in the occlusive region (287 µm) was greater than in the non-occlusive region (232 µm). Six months after IVR injection, the CBF ratio was 0.78 (9.5/12.2), and no significant difference in CT was found between the occlusive (225 µm) and non-occlusive regions (221 µm). At 12 months, the CBF ratio was 1.32 (14.6/11.1), and the CT in the occlusive region (231 µm) was slightly greater than in the non-occlusive region (218 µm). 

### 3.7. Safety

No serious adverse effects associated with IVR were observed in patients throughout the 12-month follow-up period.

## 4. Discussion

In the current study, the occlusive/non-occlusive region CBF ratio, logMAR VA, and CFT at baseline were each significantly associated with the number of IVR injections during a 12-month follow-up period in patients with treatment-naive BRVO. Choroidal and retinal thicknesses at baseline were greater in the occlusive region than in the non-occlusive region. The mean CT in the occlusive region was significantly reduced after IVR, as was the mean retinal thickness, and there was no significant change in CBF over the 12-month period. Several previous studies have reported predictive factors associated with the recurrence of ME [6,23,24,25]. Hasegawa et al. [6] recently reported that leakage patterns in FA images and subfoveal CT may be associated with response to ranibizumab therapy over a 6-month period.

Another report demonstrated that increased CFT, lower baseline VA, and shorter occlusion distance from the disc were associated with refractory ME [26]. With regard to ocular circulation, Okamoto et al. [27] reported that changes in CBF may be a useful predictor of ME recurrence associated with BRVO over a 2-month period after IVR injection. However, only a few studies have investigated the correlation between CBF and ME recurrence associated with treatment-naive BRVO over long follow-up periods. In agreement with the previous study [26], the mean number of IVR injections was significantly correlated with the logMAR VA and CFT at baseline in this study, as determined by Pearson’s correlational analysis. However, a new finding of our study was that the occlusive/non-occlusive region CBF ratio prior to therapy was significantly correlated with the mean number of IVR injections.

Generally, CBF has an important role in supplying oxygen and nutrients to the photoreceptor and RPE cells [28]. Fluid from the subretinal space is transported across the RPE cells toward the choriocapillaris [29], and a decrease in CBF induces an impairment of fluid transport due to dysfunction of the RPE cells. Luksch et al. [30] reported that the CBF increases (via a local mechanism) in eyes with BRVO to compensate for the ischemic retina. In light of the above clinical findings and the fact that the severity and extent of vascular occlusion are related to a poor response to ranibizumab [24], multiple recurrences of ME are more likely to occur in eyes with BRVO with a lower occlusive/non-occlusive region CBF ratio (i.e., a CBF imbalance). Thus, this parameter may be a useful indicator in treating ME secondary to treatment-naive BRVO.

In the current study, the mean CT was greater in the occlusive region than in the non-occlusive region at baseline, as was retinal thickness in each region. Furthermore, the mean CT in the occlusive region was significantly positively correlated with the mean retinal thickness in the occlusive region. There are several previous reports of changes in CT in patients with ME secondary to BRVO [5,10,31]. Kim et al. [10] have reported that CT was thicker in the occlusive region than in the non-occlusive region. With regard to CT in patients with treatment-naive BRVO, our results are consistent with that of the previous study. VEGF expression can reportedly influence both the outer retina and the choroidal vessels produced by ischemic regions [32,33]. Considering the above-described clinical findings, the mechanism of the thickened choroid may be mainly related to the up-regulation of VEGF in the ischemic retina of the occlusive region.

In the present study, the mean CT in the occlusive region was significantly reduced 1 week after IVR without affecting CBF. In addition, CBF in the three regions did not differ significantly from baseline at any time-point during the 12-month follow-up period. In contrast to those results, Nitta et al. [8] previously reported that CBF in the subfoveal region was reduced 1 week after intravitreal bevacizumab administration. In another previous report, CBF was significantly reduced in the subfoveal region 1 week and 1 and 2 months after IVR injection in the “good therapeutic response” group, whereas there was no significant reduction 1 and 2 months after IVR in the “poor therapeutic response” group [25]. Considering these findings, several IVR injections might have little effect on CBF in eyes with BRVO over a 12-month period. However, the reason why CBF remained unchanged after IVR injections despite a reduction in CT is unclear, and further studies are needed to clarify the mechanism involved.

This study had several limitations. First, the results were obtained from a small sample size. Further validation studies with more subjects are needed to evaluate the time-course of CBF after treatment with other anti-VEGF agents, such as aflibercept. Second, the mechanism by which the area of choroid tissues was reduced after IVR injection was not elucidated. Sonoda et al. [34] reported that the Niblack binarization method of OCT image analysis is useful for assessing the ratio of luminal and stromal areas of the choroid. To clarify the changes in the luminal area before and after IVR injection, further studies with more patients and studies investigating the luminal/stromal ratio using this method are needed. Third, nearly all patients were taking additional oral medications, such as angiotensin II receptor blockers, calcium channel blockers, statins, and hypoglycemic agents. Further studies are needed to investigate if and how these drugs affect CBF. Lastly, despite significant reductions in choroidal thickness following IVR injections, no corresponding changes in choroidal blood flow (CBF) were observed. This phenomenon may be attributed to the autoregulatory capacity of the choroid, which allows for stable blood flow despite structural alterations [35]. However, further research is needed to clarify the mechanisms underlying this discrepancy.

## 5. Conclusions

In summary, CT in the occlusive region reduced significantly after IVR injection for the entire 12-month study period, while CBF remained unchanged. An imbalance in CBF prior to therapy may be an indicator of ME recurrence after IVR injection in patients with BRVO.

## Figures and Tables

**Table 1:** Clinical and laboratory characteristics of the 16 study subjects (Mean ± SD).

Patients (*n*)	16
Men:women	10:6
Age (years)	67.6 ± 13.2
Major BRVO:macular BRVO	10:6
Axial length (mm) in the affected eye	23.0 ± 0.9
Systolic blood pressure (mmHg)	139.0 ± 21.2
Diastolic blood pressure (mmHg)	82.3 ± 11.4
Ocular perfusion pressure (mmHg)	56.5 ± 9.2
Hypertension (%)	13/16 (81.3)
Hyperlipidemia (%)	7/16 (43.8)
Type 2 diabetes mellitus (%) Administration of ARB (%) Administration of CCB (%) Administration of statin (%) Administration of hypoglycemic agents (%)	1/16 (6.3) 8/16 (50.0) 3/16 (18.8) 3/16 (18.8) 1/16 (6.3)
Duration of visual disturbance from onset (weeks)	7.8 ± 8.0
Triglyceride (mg/dL)	173 ± 218
HDL cholesterol (mg/dL)	50.9 ± 16.5
LDL cholesterol (mg/dL)	112.0 ± 18.3
Fasting plasma glucose (mg/dL)	109.0 ± 28.5
Hemoglobin A1c (%)	5.7 ± 0.3
eGFR (mL/minutes per 1.73 m^2^)	64.4 ± 22.1
Creatinine (mg/dL)	0.8 ± 0.2
Red blood cells (×10^6^ μL)	4.3 ± 0.4
Hemoglobin (g/dL)	13.7 ± 1.3
Hematocrit (%)	40.4 ± 3.4

Data are expressed as the mean ± standard deviation. BRVO, branch retinal vein occlusion; ARB, angiotensin II receptor blocker; CCB, calcium channel blocker; HDL, high-density lipoprotein; LDL, low-density; lipoprotein; eGFR, estimated glomerular filtration rate.

**Table 2:** Univariate correlation between each parameter and the number of intravitreal ranibizumab injections for the recurrence of macular edema.

			r	*p*-Value
Age			0.036	0.894
Axial length			0.154	0.615
LogMAR visual acuity at baseline			0.589	0.016
Duration of visual disturbance from onset			−0.035	0.197
Systolic blood pressure at baseline			−0.644	0.063
Diastolic blood pressure at baseline			−0.443	0.113
Ocular perfusion pressure at baseline			−0.517	0.059
Retinal thickness in the occlusive region at baseline			0.466	0.069
Retinal thickness in the non-occlusive region at baseline			−0.167	0.536
Central foveal thickness at baseline			0.697	0.013
Choroidal thickness in the occlusive region at baseline			0.151	0.576
Choroidal thickness in the non-occlusive region at baseline			−0.307	0.248
Choroidal thickness in the subfovea at baseline			−0.302	0.256
Occlusive:non-occlusive region choroidal thickness ratio at baseline			0.317	0.232
Occlusive:non-occlusive region choroidal blood flow ratio at baseline			−0.501	0.048
LogMAR, logarithm of minimal angle of resolution.				

## Data Availability

Data are available upon reasonable request.
